# CO2 delivery techniques in mini-sternotomy surgery and neurological events: a multicentric study

**DOI:** 10.1186/s12872-024-04237-8

**Published:** 2024-10-22

**Authors:** Luca P. Weltert, Katia Audisio, Michele La Torre, Michele Dell’Aquila, Gianmarco Cancelli, Vittoria Lodo, Tulio Caldonazo, Camilla S. Rossi, Giovanni J. Soletti, Luigi Garufi, Paolo Centofanti, Ruggero De Paulis, Mauro Rinaldi

**Affiliations:** 1 Heart Surgery Unit, European Hospital, Rome, Italy; 2 Department of Heart Surgery, Saint Camillus International University of Health and Medical Sciences, Rome, Italy; 3 Department of Anesthesia, Intensive Care, and Emergency, Città della Salute e della Scienza Hospital, Turin, Italy; 4 Heart Surgery Unit, San Giovanni Battista Hospital – Molinette, Turin, Italy; 5 Department of Cardiothoracic Surgery, Weill Cornell Medicine, New York City, NY USA; 6 Heart Surgery Unit, Mauriziano Hospital, Turin, Italy; 7 Department of Cardiothoracic Surgery, Friedrich-Schiller-University, Jena, Germany; 8 Department of Cardiothoracic Surgery, Catharina Ziekenhuis, Eindhoven, Netherlands

**Keywords:** Mini-sternotomy, Carbon dioxide, Air embolism, Postoperative neurological events

## Abstract

**Background:**

The impact of air bubbles into the cerebral circulation after open heart surgery has been a topic of discussion since the introduction of the heart-lung machine. The aim of the study was to evaluate whether the use of a dedicated commercial sponge diffuser is better than a custom-made narrow section cannula or the absence of CO2 in preventing neurological events after aortic valve replacement via J mini-sternotomy.

**Methods:**

Three cohorts of J-shaped mini-sternotomy performed at three different centers were prospectively compared: CO2 supplied via sponge diffuser, CO2 supplied via cannula, and no CO2 supply. Propensity matching was used to obtain comparable groups. The primary endpoints were postoperative stroke, transitory ischemic attack, convulsions, and dizziness. Secondary endpoints were 30-day mortality, duration of mechanical ventilation, and intensive care unit length of stay.

**Results:**

275 patients were enrolled in the study. After propensity matching, the sponge diffuser cohort had a significantly lower duration of mechanical ventilation (*P* < 0.001) and 30-day mortality (*P* = 0.05) when compared to the cannula cohort and the no-CO2 cohort, a lower incidence of all neurological events (*P* = 0.03) and dizziness (*P* = 0.05) when compare to the no-CO2 cohort, and a lower intensive care unit length of stay when compared to the cannula cohort (*P* = 0.001).

**Conclusions:**

The sponge diffuser used to deliver the CO2 into the surgical field during aortic valve replacement via J mini-sternotomy has been demonstrated to guarantee better neurological outcomes compared to a custom-made narrow section cannula or the absence of CO2.

## Introduction

The impact of air bubbles into the cerebral circulation after open heart surgery has been a topic of discussion since the introduction of the heart-lung machine.

Systemic air embolism occurs frequently during open heart surgery, especially after the declamping of the aorta in conjunction with the cessation of cardiopulmonary bypass [1–3]. Indeed, transcranial doppler studies have revealed that large amounts of cerebral micro-embolic signals are recorded when the heart starts to eject blood [4, 5].

Flooding the surgical field with carbon dioxide (CO2) reduces the amount of intra-cardiac air by approximately 85% and allows for a reduction in size of microemboli [6–8]. This proved to have direct impact on the amount of bubbles observed into the cerebral circulation after aortic declamping [9]. However, the advantages offered by the use of CO2 depend also on the way it is delivered into the operating field: the use of a dedicated device, pouring the CO2 into the chest without turbulence, resulted in a nearly CO2 saturated field [10, 11]. 

The lack of commercially available gas diffuser small enough to fit inside a mini-sternotomy slowed the adoption of gas diffusers in this type of surgery until 2018 [12]. In early clinical studies, the use of proper CO2 delivery techniques led to fewer gaseous microemboli [9], however, no study up to date has demonstrated beneficial clinical effects [12, 13]. 

The aim of the study was to evaluate whether the use of a dedicated commercial sponge diffuser is better than a custom-made narrow section cannula or the absence of CO2 in preventing neurological events after aortic valve replacement via J mini-sternotomy.

## Methods

### Patients

Patients undergoing aortic valve replacement via J mini-sternotomy were prospectively enrolled into an observational study at three high-volume high-expertise heart surgery centers in Italy: the European Hospital in Rome, the Mauriziano Hospital in Turin, and the San Giovanni Battista Hospital in Turin. Each hospital had established internal protocol regarding CO2 delivery in the surgical field: the European Hospital used a dedicated commercial sponge diffuser, the Mauriziano Hospital a custom-made narrow section cannula, and the San Giovanni Battista Hospital did not use CO2. All centers involved applied the same Heart Lung Machines model. The delivery of CO2 was started systematically at the time of heparin administration and discontinued immediately after declamping the aorta.

The study was approved by the local ethics committees and all patients gave informed consent.

### Propensity matching

A propensity matching by best neighbor method was applied to obtain comparable samples of the three cohorts consisting in 72 patients for each strategy. Selected variables were age, sex, hypertension, diabetes mellitus, obesity (defined as body mass index ≥ 30 kg/m^2^), chronic obstructive pulmonary disease, creatinine level > 2 mg/dl, ejection fraction, heart failure (defined as ejection fraction < 30%), pulmonary pressure, peripheral arteriopathy, preoperative atrial fibrillation, preoperative pacemaker, previous neurological events, EUROSCORE II, PARSONNET score, cardiopulmonary bypass time, and cross-clamp time.

### Endpoints

Four neurological events were recorded after surgery as primary endpoints: stroke, transitory ischemic attack (TIA), convulsions, and dizziness. Stroke was defined after instrumental imaging and neurological counseling. The timeframe limit for all four events was 48 h post-surgery. This allowed to perform a thorough neurological evaluation at least two times for every patient. Other neurological events such as postoperative delirium, excitation, confusion, and cognitive dysfunction were not included in the study due to inherent difficulty in standardization among different centers. Thirty-day mortality, duration of mechanical ventilation in hours, and intensive care unit (ICU) length of stay in days were recorded as secondary endpoints.

### Statistical analysis

The study sample size was calculated to achieve 80% statistical power at a significant level of 0.05. Calculations were based on previous not-randomized reports of short-term clinical neurological events in literature [4, 7, 14, 15]. The cohorts were powered to detect a drop in incidence from 8 to 2% in primary endpoint for dichotomous variables in independent study groups. Data collection was made from hospital datasets and entered into a dedicated spreadsheet for the analysis. Categorical variables were presented as numbers and percentages and analyzed by Pearson’s χ2 or Fisher’s exact test. Continuous variables were expressed as mean and standard deviation (SD) or median and interquartile range. Normality of the data was assessed using Shapiro-Wilk test. Differences between groups were compared using the Student’s test for normally distributed continuous variables and Mann-Whitney U test for non-normally distributed continuous variables. Analysis of variance (ANOVA) was used to compare continuous variables across > 2 groups. To obtain a proper sample matching among three cohorts, we applied the technique previously published by our group [16]. Briefly, a binary logistic regression was performed including preoperative and intraoperative variables to calculate propensity score and best neighbor matching technique was used to obtain three groups. Efficacy of the matching process was assessed repeating the regression on the extracted patients group.

P-values of < 0.05 were considered statistically significant. SPSS (version 21, IBM Corporation, Somers, New York) and Excel (Microsoft, Redmond, Washington) were used for data analysis.

## Results

From January 2018 to February 2020, 275 patients undergoing aortic valve replacement via J mini-sternotomy were enrolled in the analysis. One hundred and thirteen patients from the San Giovanni Battista Hospital, 82 from the European Hospital, and 80 from the Mauriziano Hospital. Preoperative and intraoperative data are summarized in Table 1. Patients in the sponge diffuser cohort were older when compared to the cannula cohort (73.3 ± 8.9 vs. 69.3 ± 9.9, *P* = 0.01) or the no-CO2 cohort (73.3 ± 8.9 vs. 67.5 ± 10.3, *P* = 0.001) and had a lower ejection fraction (52.3 ± 7.9 vs. 59.7 ± 8.8 vs. 57.2 ± 8.2, *P* < 0.001). The extracorporeal circulation time was lower in the sponge diffuser cohort when compared to the cannula cohort (81.0 ± 23.0 min vs. 98.4 ± 29.0 min, *P* = 0.001) or the no-CO2 cohort (81.0 ± 23.0 min vs. 90.0 ±.

**Table 1:** Baseline and intraoperative characteristics in the raw cohorts

RAW DATA	Sponge diffuser (*n* = 82)		Cannula (*n* = 80)		No-CO2 (*n* = 113)			*P* Sponge vs. Cannula		*P* Sponge vs. no-CO2	*P* Cannula vs. no-CO2
	N or Mean	% or SD	N or Mean	% or SD		N or Mean	% or SD				
Age	73.3	8.9	69.3	9.9		67.5	10.3		**0.01**	**0.001**	0.2
Male sex	50	61	58	63.7		77	68.1		0.8	0.4	0.6
Hypertension	76	92.7	78	97.5		110	97.4		**0.05**	0.5	0.3
Diabetes Mellitus	16	19.5	17	18.7		23	20.4		1.0	1.0	0.9
Obesity	5	6.1	4	4.4		6	5.3		0.7	1.0	1.0
COPD	4	4.9	6	6.6		3	2.7		0.8	0.5	0.3
Creatinine > 2 mg/dl	2	2.4	1	1.1		2	1.8		0.6	1.0	1.0
Ejection Fraction (%)	52.3	7.9	59.7	8.8		57.2	8.2		**< 0.001**	**< 0.001**	**0.03**
Heart Failure	0	0	3	3.3		0	0		0.2	1.0	0.09
Pulmonary Pressure 30–60 mmHg	4	4.9	3	3.3		4	3.5		0.7	0.7	1.0
Pulmonary pressure > 60mmHg	0	0	1	1.1		0	0		1.0	1.0	0.4
Critical Carotid Artery Stenosis	0	0	0	0		1	0.9		1.0	1.0	1.0
Peripheral Arteriopathy	6	7.3	6	6.6		3	2.7		1.0	0.2	0.03
Preoperative Atrial Fibrillation	3	3.7	7	7.7		9	8		0.3	0.2	1.0
Preoperative Pacemaker	3	3.7	1	1.1		2	1.8		0.3	0.7	1.0
Previous Neurological Events	4	4.9	2	4.4		9	8		1.0	0.6	0.6
EUROSCORE II	7.7	5.5	6.1	7.8		2.6	1.2		0.1	**< 0.001**	**< 0.001**
PARSONNET score	1.9	2.1	1.8	2.6		1.2	1.8		0.8	**0.01**	0.1
Mechanical Valve Implant	0	0	1	1.1		15	13.3		1.0	**0.002**	**0.001**
Surgery on Ascending Aorta	0	0	3	3.3		0	0		0.2	1.0	0.1
CPB Time	81	23	98.4	29		90	24		**0.001**	**0.02**	0.1
Cross-Clamp Time	46	13	69.3	17.8		54	15		**< 0.001**	**0.001**	**< 0.001**

COPD: chronic obstructive pulmonary disease; CPB: cardiopulmonary bypass; IABP: intra-aortic balloon pump

Statistically significant p values are in bold.

24.0, *P* = 0.02) as well as the cross-clamp time (46.0 ± 13.0 vs. 69.3 ± 17.8 vs. 54.0 ± 15.0, *P* ≤ 0.001).

After the propensity matching, each cohort consisted of 72 patients with comparable characteristics. Preoperative and intraoperative data for the propensity matched cohorts are provided in Table 2.

**Table 2:** Baseline and intraoperative characteristics in the propensity-matched cohorts

MATCHED DATA	Sponge diffuser (*n* = 72)		Cannula (*n* = 72)		No-CO2 (*n* = 72)		*P* Sponge vs. Cannula	*P* Sponge vs. no-CO2	*P* Cannula vs. no-CO2
	N or Mean	% or SD	N or Mean	% or SD	N or Mean	% or SD			
Age	70.3	9.9	69.3	8.7	67.5	9.2	0.5	0.1	0.2
Male sex	56	77.8	56	77.8	58	80.6	1.0	0.8	0.8
Hypertension	70	97.2	71	98.6	69	95.8	1.0	1.0	0.6
Diabetes Mellitus	15	20.8	16	22.2	14	19.4	1.0	1.0	0.8
Obesity	4	5.6	4	5.6	5	6.9	1.0	1.0	1.0
COPD	4	5.6	4	5.6	4	5.6	1.0	1.0	1.0
Creatinine > 2 mg/dl	1	1.4	1	1.4	1	1.4	1.0	1.0	1.0
Ejection Fraction (%)	52.3	8	53	7.8	54	8.1	0.6	0.2	0.5
Heart Failure	0	0	1	1.4	0	0	1.0	1.0	1.0
Pulmonary Pressure 30–60 mmHg	3	4.2	3	4.2	3	4.2	1.0	1.0	1.0
Peripheral Arteriopathy	3	4.2	3	4.2	3	4.2	1.0	1.0	1.0
Preoperative Atrial Fibrillation	3	4.2	3	4.2	3	4.2	1.0	1.0	1.0
Preoperative Pacemaker	1	1.4	1	1.4	1	1.4	1.0	1.0	1.0
Previous Neurological Events	3	4.2	3	4.2	3	4.2	1.0	1.0	1.0
EUROSCORE II	7.1	6.8	6.7	6.1	6.9	6.2	0.7	0.9	0.8
PARSONNET score	1.9	2.6	1.9	2.1	1.9	2.3	1.0	0.9	0.9
CPB Time	86	25	90	25	92	26	0.3	0.3	0.6
Cross-Clamp Time	53	14	58	19	58	17	0.1	0.1	1.0

COPD: chronic obstructive pulmonary disease; CPB: cardiopulmonary bypass; IABP: intra-aortic balloon pump

### Primary endpoints

In the raw cohorts, there were no differences in the primary outcomes between sponge diffuser vs. cannula vs. no-CO2 for stroke (*n* = 0/82 [0.0%] vs. 2/80 [2.5%] vs. 2/113 [1.8%]) and dizziness (*n* = 0/82 [0.0%] vs. 1/80 [1.3%] vs. 5/113 [4.4%]). Only 1 patient (0.9%) in the no-CO2 cohort experienced a TIA and only 1 patient (1.2%) in the sponge diffuser cohort experienced convulsions (Table 3).

**Table 3:** Outcomes in the raw cohorts

RAW DATA	Sponge diffuser (*n* = 82)		Cannula (*n* = 80)		No-CO2 (*n* = 113)		*P* Sponge vs. Cannula	*P* Sponge vs. no-CO2	*P* Cannula vs. no-CO2
	N or Mean	% or SD	N or Mean	% or SD	N or Mean	% or SD			
All neurological events	1	1.2	3	3.8	8	7.1	0.2	0.1	0.8
Stroke	0	0	2	2.5	2	1.8	0.5	0.5	1.0
TIA	0	0	0	0	1	0.9	1.0	1.0	1.0
Convulsions	1	1.2	0	0	0	0	1.0	1.0	1.0
Dizziness	0	0	1	1.3	5	4.4	0.2	0.1	0.7
30-day mortality	0	0	1	1.3	1	0.9	0.1	1.0	0.1
Duration of mechanical ventilation (h)	5.5	3.9	11.6	9.2	15.5	5.9	< 0.001	< 0.001	0.01
ICU length of stay (d)	1.2	2.2	2	2	1.5	3.1	0.01	0.5	0.1

ICU: intensive care unit; TIA: transient ischemic attack

After propensity matching, the sponge diffuser cohort had a significantly lower incidence of all neurological events (*n* = 1/72 [1.4%] vs. 8/72 [11.1%], *P* = 0.03) and dizziness (*n* = 0/72 [0%] vs. 5/72 [6.9%], *P* = 0.05) when compared to the no-CO2 cohort. For stroke (*n* = 0/72 [0%] vs. 2/72 [2.8%] vs. *n* = 2/72 [2.8%]), TIA (*n* = 0/72 [0%] vs. 0/72 [0%] vs. *n* = 1/72 [1.4%]), and convulsions (*n* = 1/72 [1.4%] vs. 0/72 [0%] vs. 0/72 [0%]) no differences were reported between sponge diffuser vs. cannula vs. no-CO2 (Table 4).

**Table 4:** Outcomes in the propensity-matched cohorts

MATCHED DATA	Sponge diffuser (*n* = 72)		Cannula (*n* = 72)		No-CO2 (*n* = 72)		*P* Sponge vs. Cannula	*P* Sponge vs. no-CO2	*P* Cannula vs. no-CO2
	N or Mean	% or SD	N or Mean	% or SD	N or Mean	% or SD			
All neurological events	1	1.4	3	4.2	8	11.1	0.2	0.03	0.5
Stroke	0	0	2	2.8	2	2.8	0.5	0.5	1.0
TIA	0	0	0	0	1	1.4	1.0	1.0	1.0
Convulsions	1	1.4	0	0	0	0	1.0	1.0	1.0
Dizziness	0	0	1	1.4	5	6.9	0.2	0.05	0.7
30-day mortality	0	0	1	1.4	1	1.4	0.05	0.05	0.2
Duration of mechanical ventilation (h)	6.2	3.3	9.6	6.7	13.3	5.4	< 0.001	< 0.001	0.002
ICU length of stay (d)	1.2	1,4	2.2	2.1	1.7	2.7	0.001	0.2	0.2

ICU: intensive care unit; TIA: transient ischemic attack

### Secondary endpoints

In the raw cohorts, there was no difference in 30-day mortality between sponge diffuser vs. cannula vs. no-CO2 (*n* = 0/82 [0.0%] vs. 1/80 [1.3%]] vs. 1/113 [0.9%]). Duration of mechanical ventilation was lower in the sponge diffuser cohort when compared to the cannula cohort (5.5 ± 3.9 h vs. 11.6 ± 9.2, *P* < 0.001) or the no-CO2 cohort (5.5 ± 3.9 h vs. 15.5 ± 5.9 h, *P* < 0.001). ICU length of stay was lower in the sponge diffuser cohort when compared to the cannula cohort (1.2 ± 2.2 days vs. 2.0 ± 2.0 days, *P* = 0.01).

In the propensity matched cohorts, 30-day mortality rate was higher in the cannula cohort and in the no-CO2 cohort when compared to the sponge diffuser cohort (1.4% vs. 1.4% vs. 0% respectively, *P* = 0.05). Duration of mechanical ventilation was lower in the sponge diffuser cohort when compared to the cannula cohort (6.2 ± 3.3 h vs. 9.6 ± 6.7 h, *P* < 0.001) or the no-CO2 cohort (6.2 ± 3.3 h vs. 13.3 ± 5.4 h, *P* < 0.001). ICU length of stay was lower in the sponge diffuser cohort when compared to the cannula cohort (1.2 ± 1.4 days vs. 2.2 ± 2.1 days, *P* = 0.001).

Further stratification in the sponge diffuser cohort revealed that all events occurred to patients in which a CO2 flow of 2.5 l/min was used, while no patient with a CO2 flow of 5 l/min experienced neurological events.

## Discussion

In the present analysis, a dedicated commercial sponge diffuser used to deliver CO2 into the surgical field during aortic valve replacement via J mini-sternotomy seems to guarantee better neurological outcomes compared to a custom-made narrow section cannula or the absence of CO2. The gas mixture into the mediastinum is certainly not the only factor involved in the creation of microemboli; for instance, the type of lung perfusion and ventilation during extracorporeal circulation also represents an important factor, as well as the cardiopulmonary bypass suction vent.

However, taking into consideration these inherent limitations, the sponge diffuser cohort had a significantly lower duration of mechanical ventilation and 30-day mortality when compared to the cannula cohort and the no-CO2 cohort, a lower incidence of all neurological event and dizziness when compared to the no-CO2 cohort, and a lower ICU length of stay when compared to the cannula cohort. Moreover, in the sponge diffuser cohort, all events occurred to patients in which a CO2 flow of 2.5 l/min was used, while no patient with a CO2 flow of 5 l/min experienced neurological events.

Due to its higher blood solubility, CO2 is better than air in preventing micro-bubbles formation when displaced into the operating field during open heart surgery [17]. 

The air removal process, known as de-airing, is a fundamental part of the operation performed prior to the cross-clamp removal. Various de-airing techniques have been proposed to minimize air micro-embolization during open heart surgery, such as filling and venting of the left ventricle, heart and lungs’ ballottement, mechanical ventilation, Trendelenburg position, and needle aspiration [1, 3]. Despite these efforts, a significant amount of air remains in the heart cavities [9], especially in minimally invasive context in which the de-airing maneuvers might be impervious. In earlier clinical studies, the use of CO2 led to fewer gaseous microemboli, with total de-airing of heart cavities occurring 7 min after cross-clamp removal versus 17 min for patients in which CO2 was not adopted [9]. 

The advantages of CO2, however, do not only depend on the intrinsic characteristics of the molecule but also on how it is delivered into the operating field. Several studies have showed a difference in the final content of residual air into the surgical field based on the CO2 delivery system adopted: a long and narrow cannula generates an accelerate flow that leads to a turbulent mixing of CO2 and air molecules with an average CO2 concentration in the field around 50%.^10,11^ On the other hand, the soft flooding granted by a dedicated diffuser led to a nearly complete air displacement obtaining a uniform CO2 concentration around 99%.^10,11^ These differences have been proved to directly impact on the amount of bubbles observed into the cerebral circulation after aortic declamping [9]. 

The discussion about causal correlation between peri-procedural microemboli and neurocognitive events has raged in the first decade of the millennium and is nowadays accepted a multifactorial etiology of the phenomenon, with cerebral hypoperfusion and systemic inflammatory response playing a role in the physiopathology of neurological complications after open heart surgery [18]. In addition, the type of anesthesia, blood loss, postoperative pain management strategy, for instance, represent, potentially, all significant factors and thus, should be isolated and considered singularly. As we did not acquire these variables at the time of the stud, this may contribute to a bias. Previous studies evaluating the effect of CO2 delivery on neurocognitive outcomes reported controversial results [15, 19]. Furthermore, these studies only adopted neurocognitive function tests to analyze the outcomes and it is now widely recognized that such tests may lack precision [20]. This is why we focused on relatively unmistakable events when assessing the efficacy of the techniques and limit our observation to the very early postoperative phase.

Our study suffered first and foremost from the limitations of a multicentric non-randomized study, therefore unknown confounding factors might influence some of the results despite the propensity matching. Additionally, the study was limited by the small number of patients given the low incidence of neurological complications after open heart surgery and might benefit from additional recruitment of patients. Furthermore, some of the events might be dependent on non-gaseous embolic etiology, but no actual diagnostic tool can be used to discriminate. Mortality, albeit statistically different, is a multifactorial endpoint that cannot be exclusively connected to the CO2 delivery technique; duration of mechanical ventilation, on the other hand, might be connected to an earlier awakening of the patients due to fewer microemboli, or could be simply due to the choice of the anesthesiologist. At least it can be stated that the sponge diffuser is not associated with detrimental effects reflecting in longer duration of mechanical ventilation or higher mortality, which means that, apart from the financial burden, it does not inflict any apparent harm, while potentially being beneficial.

## Conclusions

To conclude, the novelty of this study is that a dedicated commercial sponge diffuser used to deliver the CO2 into the surgical field during aortic valve replacement via J mini-sternotomy seems to guarantee better neurological outcomes compared to a custom-made narrow section cannula or the absence of CO2. The sponge diffuser might be the preferred technique to obtain proper displacement of air from the operating field during open heart surgery.

## Data Availability

The datasets used and/or analyzed during the current study available from the corresponding author on reasonable request.

## Abbreviations

COPD: Chronic Obstructive Pulmonary Disease
ECC: Extracorporeal Circulation
IABP: Intra-aortic Balloon Pump
ICU: Intensive Care Unit
SD: Standard Deviation
TEE: Transesophageal Echocardiography
TIA: Transient Ischemic Attack
